# Differential care-seeking behaviors during the beginning of the COVID-19 pandemic in Michigan: a population-based cross-sectional study

**DOI:** 10.1186/s12889-023-16999-5

**Published:** 2023-10-25

**Authors:** Catherine A. Vander Woude, Elizabeth J. King, Jana L. Hirschtick, Andrea R. Titus, Laura E. Power, Michael R. Elliott, Nancy L. Fleischer

**Affiliations:** 1 Department of Epidemiology, University of Michigan School of Public Health, Ann Arbor, MI USA; 2 Department of Health Behavior and Health Education, University of Michigan School of Public Health, Ann Arbor, MI USA; 3 Department of Population Health, Grossman School of Medicine, New York University, New York, NY USA; 4 Department of Biostatistics, University of Michigan School of Public Health, Ann Arbor, MI USA; 5 Survey Research Center, Institute for Social Research, University of Michigan, Ann Arbor, MI USA

**Keywords:** COVID-19, Access to care, Healthcare utilization

## Abstract

**Background:**

At the beginning of the COVID-19 pandemic in the United States in the spring of 2020, many Americans avoided the healthcare system, while those with COVID-19 symptoms were faced with decisions about seeking healthcare services for this novel virus.

**Methods:**

Using a probability sample (n = 1088) from the Michigan adult population of PCR-confirmed COVID-19 cases who were diagnosed prior to July 31, 2020, we used logistic regression to examine sociodemographic and symptom severity predictors of care-seeking behaviors. The analyses examined three different outcomes: (1) whether respondents sought care and, among those who sought care, whether they sought care from (2) a primary care provider or (3) an emergency room. Final models were adjusted for sex, age, race and ethnicity, income, education, marital status, living arrangement, health insurance, and self-reported symptom severity.

**Results:**

We found that participants ages 65 and older had 4.00 times higher odds of seeking care than 18-34-year-olds (95% CI: 2.21, 7.24), while adults reporting very severe symptoms had roughly 15 times higher odds of seeking care than those with mild symptoms (95% CI: 7.73, 27.01). Adults who were non-Hispanic Black or were uninsured had lower odds of seeking care from a primary care physician versus seeking care from other locations in comparison to adults who were non-Hispanic White or were privately insured, respectively (non-Hispanic Black: aOR = 0.27, 95% CI: 0.16, 0.44; Uninsured: aOR = 0.19, 95% CI: 0.09, 0.42). Conversely, adults who were older or reported more severe symptoms had higher odds of seeking care from an emergency room versus other locations in comparison to adults who were younger or reported less severe symptoms (Age 65+: aOR = 2.96, 95% CI: 1.40, 6.28; Very Severe Symptoms: aOR = 6.63, 95% CI: 3.33, 13.20).

**Conclusions:**

Our results suggest differential utilization of healthcare services early in the COVID-19 pandemic. Further analyses are needed to examine the reasons for these differences.

**Supplementary Information:**

The online version contains supplementary material available at 10.1186/s12889-023-16999-5.

## Background

The beginning of the COVID-19 pandemic brought a rapidly changing landscape for people seeking medical care. Prior research has found that overall healthcare utilization rates declined significantly when COVID-19 first became widespread in the United States in March 2020. As of June 2020, over 40% of adults in the U.S. avoided the healthcare system due to COVID-19 concerns [1]. Additionally, emergency room (ER) usage dropped by 42% in April 2020 in comparison to April 2019, while primary care and specialist visits declined by nearly 60% between March and April of 2020 [2, 3]. During a pandemic, it is essential that the healthcare system be prepared for changes in care-seeking patterns due to the public’s heightened concern about infection. One way to do this is to examine the predictors of seeking care for both historical outbreaks, as well as day-to-day health needs, in order to make the most informed decisions about preparedness policies and resource allocation.

Sociodemographic predictors of care-seeking behavior have been identified in the literature for both prior pandemics and day-to-day health needs. In 2009, during the H1N1 influenza pandemic in the U.S., women and adults ages 65 and older were more likely to seek healthcare for their influenza-like illness symptoms than men and adults ages 18 to 64 years old, respectively [4, 5]. In contrast, adults who did not have health insurance or reported financial barriers to accessing healthcare were less likely to seek healthcare than their counterparts [4]. Research on H1N1 and severe acute respiratory syndrome (SARS) showed that there was an increase in care-seeking behavior during these pandemics compared to normal times [6]. Furthermore, individual perception of disease severity was cited as a significant predictor of seeking care [6]. Additional research focusing on care-seeking behaviors during a typical influenza season found that at least half of both U.S. adults and adults in other countries with influenza-like illness did not seek healthcare for their symptoms [5–7]. One U.S. study examined where individuals sought care during a typical influenza season with roughly half of participants who sought care doing so at a doctor’s office and less than 10% doing so at an emergency room [5]. Examining trends like these for the COVID-19 pandemic may help healthcare systems efficiently allocate resources across points of care during times of heightened risk perception among the public. However, day-to-day trends may also help elucidate these care-seeking trends.

Sociodemographic predictors of care-seeking behaviors for day-to-day needs have been studied for decades. Research on day-to-day care utilization has shown that married people are more likely than unmarried people to visit healthcare providers, but are less likely than unmarried people to utilize the ER for their healthcare needs [8]. A similar pattern can be seen in terms of age: adults ages 65 + are more likely to receive a larger portion of their care from physicians’ offices, while 20- to 29-year-olds are more likely receive more of their medical care in the ER instead of outpatient facilities [9]. Despite this, adults ages 65 + use emergency services at a rate four times that of adults under the age of 65 [10]. Higher educational attainment is also associated with more healthcare visits in general, but fewer ER visits, in comparison to lower educational attainment [8]. Race and ethnicity have also been associated with location of care: non-Hispanic Black adults are more likely than non-Hispanic White adults to utilize the ER [11]. Additionally, one of the greatest predictors of where an individual seeks care is health insurance status, which is closely tied to socioeconomic status. Compared to individuals with private insurance, Medicare beneficiaries are twice as likely to utilize the ER, while Medicaid beneficiaries are nearly four times as likely to utilize the ER [12]. Other insurance coverage factors, such as underinsurance and not being insured, have also been cited as drivers of ER usage [13].

The state of Michigan was hit particularly hard at the start of the COVID-19 pandemic in the United States. As of July 31, 2020—the end date used in our study—there had been over 160,000 confirmed cases of COVID-19 in the state of Michigan and nearly 6,300 deaths [14]. We examined sociodemographic predictors of care-seeking behavior among a population-based sample of adults with PCR-confirmed SARS-CoV-2 infection in Michigan with onset on or before July 31, 2020. The current literature examines COVID-19 care-seeking measures among select clinical [15, 16] and international populations [17–19], as well as commentaries describing the scope of access to care [20]. Thus far, relatively little is known about healthcare utilization patterns among population-based samples of people who tested positive for COVID-19 and how these patterns vary by sociodemographic factors. In the event of a future pandemic, this information may help with resource allocation and preparedness. We hypothesized that adults with lower socioeconomic status, including uninsured individuals and Medicaid recipients, will have lower odds of seeking medical care for COVID-19, but higher odds of visiting the ER when seeking care, compared to adults with higher socioeconomic status and private insurance. In determining the sociodemographic predictors of healthcare utilization during the COVID-19 pandemic, we aimed to provide evidence to support the healthcare response during future public health emergencies and to lay the groundwork for future studies examining healthcare needs during the COVID-19 pandemic.

## Methods

### Michigan COVID-19 Recovery Surveillance Study

The data for this study was collected through the Michigan COVID-19 Recovery Surveillance Study (MI CReSS), a joint effort by the University of Michigan School of Public Health and the Michigan Department of Health and Human Services to conduct surveillance of adults diagnosed with COVID-19 in Michigan [21]. MI CReSS is a population-based study that includes a probability sample of adults who are at least 18 years old and have record of a positive SARS-CoV-2 PCR test in the Michigan Disease Surveillance System (MDSS). Individuals were eligible for inclusion if they had sufficient data in the MDSS, including their name, phone number, geographic information (county/zip code), and date of birth. Individuals who were deceased or institutionalized (i.e., in psychiatric hospitals or prisons) were excluded.

This analysis includes data from the first three samples of MI CReSS. The study utilized a stratified, probability sample of eligible cases. The sampling strata included 13 geographic areas in Michigan: six emergency preparedness regions [22] (each consisting of multiple counties), six counties, and one city (Detroit) in southeast Michigan. The study sample consisted of 4,000 cases drawn from over 66,000 eligible individuals with COVID-19 onset between January 2020 and July 2020. This timeframe was chosen because it encompasses the first, big wave of COVID-19 cases in Michigan, which was a time when access to healthcare was potentially the most limited due to stay-at-home orders and general hesitancy to seek healthcare.

All individuals in the sample were mailed an introductory recruitment letter, which included a link to complete the survey online. Respondents who did not complete the survey online were called by trained interviewers who conducted interviews in English, Spanish, or Arabic. A total of 1,215 interviews were completed between June 23, 2020 and May 19, 2021, yielding a response rate of 31.8% and a cooperation rate of 51.6% [22]. To account for nonresponse as well as chance imbalances in the sample, sampling weights were constructed using generalized regression estimators [23] so that the weighted distribution of the sample matched the age and sex distribution by geographic region of the sampling frame.

After excluding cases with missing data, the analytic sample included 1088 cases. Missing data ranged from 0.2% (health insurance type) to 6.8% (living arrangement). Analyses of where individuals sought care for their COVID-19 illness were completed using a subsample containing the 677 cases who sought treatment for COVID-19. Supplementary Fig. 1, a flowchart describing the creation of the analytic sample and subsample, can be found in Additional File 1.

### Care-seeking behaviors

Our primary outcome of interest was a binary indicator of whether survey respondents sought treatment for their COVID-19 symptoms. In the MI CReSS survey, respondents were asked, “Besides testing, did you seek treatment for COVID-19 illness from any of the following places?” They were then asked to reply “yes” or “no” to the following options: primary care physician/family doctor, urgent care clinic, ER, retail clinic/pharmacy, and somewhere else (specify). Respondents were classified as having not sought care if they answered “no” to seeking care from a primary care physician/family doctor, urgent care clinic, ER, and retail clinic/pharmacy, and they either said they did not seek care from somewhere else or had missing data for the somewhere else variable. If respondents answered “yes” to any of the options, they were classified as having sought care. After examining whether respondents sought care, we further broke down the analyses to examine where respondents sought care. We used two additional binary outcomes defined among the subset of the sample that did seek care. If the respondent replied “yes” to having sought care at care from a primary care physician/family doctor (PCP) — regardless of where else they may have sought COVID-19 treatment — they were coded as having sought care from a PCP. Similarly, regardless of any other places they sought care, if a respondent answered that they had visited the ER, they were coded as having sought care from an ER. The referent group for these analyses was having sought care from somewhere other than a PCP or ER, respectively.

### Sociodemographic predictors

The sociodemographic predictors in this study included age (18–34, 35–54, 55–64, 65+), sex (male, female), race and ethnicity (Hispanic, non-Hispanic White, non-Hispanic Black, another race or ethnicity), annual household income (<$35,000, $35,000-$74,999, > $75,000), educational attainment (high school education or less, some college or technical school, college graduate), marital status (married or living with a partner in a marriage-like relationship, widowed/divorced/separated/never married), and living arrangement (owns home, rents home, other arrangement).

To account for income missingness (12.3% of the combined sample), we used the weighted sequential hot-deck (WSHD) method [24] and hot deck propensity score (HDPS) imputation [25] to impute annual household income under the missing at random assumption.

### Health insurance type

Respondents reported insurance status and type of insurance, if any, at the time of their COVID-19 illness. If an individual identified more than one type of health insurance, we applied an algorithm to determine what type of insurance was most likely to cover their COVID-19 care as their primary payer [26, 27], prioritizing private insurance, followed by Medicare, Medigap, military-related healthcare (e.g., TRICARE, CHAMPVA, VA healthcare), Medicaid, and the Indian Health Service. After applying the algorithm, the following final categories of insurance type were established to ensure adequate sample sizes: private, Medicare, Medicaid, other, and uninsured.

### Symptom severity

Respondents reported their symptom severity when symptoms were at their worst as asymptomatic, mild, moderate, severe, or very severe. Due to testing limitations early in the pandemic, only a small number of asymptomatic individuals were included early in the study, and an asymptomatic answer choice was not available in the survey tool early on in data collection. Most individuals who denied all symptoms selected the mild option. Other respondents who were missing the symptom severity variable (and denied all symptoms) were assigned to the asymptomatic/mild category so that they were included in the analysis (n = 7).

### Analysis

We performed a series of four sequentially adjusted logistic regression models for each of our outcome variables (i.e., sought care, primary care, ER). Model 1 was unadjusted; Model 2 included sociodemographic predictors; Model 3 added health insurance; and Model 4 added self-reported severity of symptoms. Unadjusted and adjusted odds ratios were reported with 95% confidence intervals. All adjusted models additionally controlled for sample and survey mode.

### Sensitivity analysis

We conducted two sensitivity analyses to further understand our results. The first was a multinomial logistic regression investigating predictors of primary care only, emergency room only, or both primary care and emergency care, compared to other locations, in order to understand the overlap of primary care and emergency care use. We created a new outcome variable defined as “Primary Care, but not Emergency Room,” “Emergency Room, but not Primary Care,” “Primary Care and Emergency Room,” and “Sought care but not from Primary Care or the Emergency Room,” and reran the regression models to examine predictors. The second sensitivity analysis adjusted for an additional covariate: pre-existing conditions. The survey asks respondents to answer “yes” or “no” to whether a doctor or health professional has ever told them that they had following comorbidities: Emphysema or chronic obstructive pulmonary disease (COPD); asthma; diabetes; heart disease; high blood pressure; liver disease, kidney disease; stroke or other cerebrovascular disease; cancer; immunosuppressive condition; autoimmune condition; physical disability; psychological/ psychiatric condition; and any other condition. The number of comorbidities for each respondent was then summed and categorized as “0 Comorbidities,” “1 Comorbidity,” “2 Comorbidities,” or “≥3 Comorbidities.” We examined the regression models additionally adjusting for comorbidities as a sensitivity analysis rather than including the covariate in the main models because, while it may be an important predictor of care-seeking behavior, it may also be a mediator between sociodemographic factors and care-seeking behavior.

SAS version 9.4 and Stata SE, version 18 were used for data analyses, including adjustment for weights and the complex survey design [28, 29]. This analysis was considered exempt by the University of Michigan Institutional Review Board (HUM00194114).

## Results

### Descriptive analysis

The study sample was majority female (56.6%), non-Hispanic White (52.1%), and younger than 55 (25.4% 18–34 years old, 40.6% 35–54 years old) (Table 1). Most respondents (71.4%) had private health insurance, while 8.6% reported not having health insurance. Roughly a fifth of respondents categorized their symptoms as very severe (21.3%), while just under a third labeled their symptoms severe (30.9%). Most respondents sought care for COVID-19 (62.7%), with a slightly greater percentage seeking care from a primary care office (36.4%) versus the ER (32.3%).

**Table 1:** Description of analytic sample (n = 1088), MI CReSS

	Weighted Percent	Frequency
Sex		
Male	43.4%	435
Female	56.6%	653
Age Group		
18 to 34	25.4%	258
35 to 54	40.6%	420
55 to 64	20.0%	228
65+	14.0%	182
Race and Ethnicity		
Hispanic	11.1%	107
Non-Hispanic White	52.1%	654
Non-Hispanic Black	24.5%	212
Another race or ethnicity	12.3%	115
Annual Household Income		
<$35,000	31.7%	329
$35,000-$74,999	29.5%	332
$75,000+	38.9%	427
Education		
High school education or less	27.4%	286
Some college or technical school	35.1%	392
College graduate	37.5%	410
Marital Status		
Widowed, divorced, separated, or never married	38.8%	405
Married or living with a partner in a marriage-like relationship	61.2%	683
Living Arrangement		
Rent	25.8%	256
Own	62.2%	704
Other arrangement	12.1%	128
Health Insurance		
Uninsured	8.6%	92
Private	71.4%	794
Medicare	8.6%	91
Medicaid	8.8%	81
Another type	2.6%	30
Self-Reported Symptom Severity		
Mild	24.1%	260
Moderate	23.7%	274
Severe	30.9%	328
Very severe	21.3%	226
Care-seeking Behavior		
Did not seek care	37.3%	411
Sought care	62.7%	677
Care Location		
Went to Primary Care Provider	36.4%	399
Went to Emergency Room	32.3%	347

### Care-seeking behavior

In all adjusted models, the odds of seeking care were greater among older age groups in comparison to 18- to 34-year-olds, with the greatest odds among respondents over the age of 65 (Table 2, Model 2: aOR = 3.95, 95% CI: 2.32, 6.73). Sex, race and ethnicity, income, education, marital status, living arrangement, and health insurance type were not associated with care-seeking in the final adjusted model. However, race and ethnicity, income, education, and health insurance type were statistically significant in the unadjusted models. In the final adjusted model, respondents with very severe symptoms had 14.45 times higher odds (Model 4: 95% CI: 7.73, 27.01) of seeking care than respondents who reported mild symptoms.

**Table 2:** Predictors of seeking care for COVID-19 using logistic regression (n = 1088), MI CReSS

	(1)		(2)		(3)		(4)	
	Odds Ratio	95% CI	Odds Ratio	95% CI	Odds Ratio	95% CI	Odds Ratio	95% CI
Sex								
Male	1.00		1.00		1.00		1.00	
Female	1.01	[0.76, 1.34]	1.04	[0.77, 1.42]	1.04	[0.76, 1.43]	0.99	[0.71, 1.38]
Age Group								
18 to 34	1.00		1.00		1.00		1.00	
35 to 54	3.65***	[2.55, 5.23]	3.34***	[2.24, 4.98]	3.36***	[2.24, 5.04]	2.91***	[1.91, 4.45]
55 to 64	3.78***	[2.47, 5.80]	3.28***	[2.03, 5.31]	3.34***	[2.06, 5.42]	2.51***	[1.49, 4.23]
65+	4.89***	[3.05, 7.84]	3.95***	[2.32, 6.73]	3.89***	[2.24, 6.77]	4.00***	[2.21, 7.24]
Race/Ethnicity								
Hispanic	1.66*	[1.02, 2.70]	1.55	[0.89, 2.69]	1.48	[0.85, 2.57]	1.14	[0.61, 2.10]
Non-Hispanic White	1.00		1.00		1.00		1.00	
Non-Hispanic Black	1.72**	[1.20, 2.47]	1.03	[0.68, 1.56]	1.03	[0.68, 1.55]	0.94	[0.60, 1.45]
Another race/ethnicity	1.61*	[1.02, 2.53]	1.59	[0.96, 2.64]	1.53	[0.92, 2.54]	1.46	[0.86, 2.50]
Annual Household Income								
<$35,000	1.36	[0.98, 1.90]	1.39	[0.90, 2.16]	1.27	[0.79, 2.02]	1.22	[0.72, 2.05]
$35,000-$74,999	1.15	[0.82, 1.61]	1.23	[0.84, 1.81]	1.22	[0.83, 1.80]	1.07	[0.70, 1.62]
$75,000+	1.00		1.00		1.00		1.00	
Education								
High school education or less	1.58*	[1.11, 2.25]	1.04	[0.68, 1.58]	1.01	[0.66, 1.54]	1.22	[0.77, 1.92]
Some college or technical school	1.15	[0.84, 1.59]	1.04	[0.72, 1.48]	1.02	[0.71, 1.46]	1.00	[0.68, 1.45]
College graduate	1.00		1.00		1.00		1.00	
Marital Status								
Widowed, divorced, separated, or never married	0.81	[0.61, 1.08]	0.94	[0.65, 1.34]	0.94	[0.65, 1.35]	0.96	[0.65, 1.42]
Married or living with a partner in a marriage-like relationship	1.00		1.00		1.00		1.00	
Living Arrangement								
Rent	0.82	[0.59, 1.13]	1.07	[0.69, 1.64]	1.02	[0.66, 1.58]	0.94	[0.60, 1.48]
Own	1.00		1.00		1.00		1.00	
Other arrangement	0.68	[0.45, 1.05]	1.09	[0.64, 1.85]	1.03	[0.60, 1.76]	0.97	[0.54, 1.72]
Health Insurance Type								
Uninsured	1.01	[0.61, 1.65]			1.43	[0.80, 2.54]	1.41	[0.77, 2.58]
Private	1.00				1.00		1.00	
Medicare	1.89*	[1.08, 3.29]			1.35	[0.71, 2.57]	1.18	[0.59, 2.37]
Medicaid	1.21	[0.72, 2.05]			1.31	[0.67, 2.54]	1.29	[0.60, 2.78]
Another Type	1.30	[0.55, 3.10]			1.14	[0.46, 2.85]	1.18	[0.46, 3.00]
Self-Reported Severity of Symptoms								
Mild	1.00						1.00	
Moderate	2.30***	[1.55, 3.42]					2.23***	[1.46, 3.42]
Severe	4.17***	[2.82, 6.17]					3.44***	[2.28, 5.19]
Very severe	19.77***	[10.87,35.97]					14.45***	[7.73,27.01]

* p < 0.05, ** p < 0.01, *** p < 0.001

The weighted population size was n = 59,837, which reflects the Michigan population with PCR-confirmed COVID-19 who sought care during this period. Model 1 is unadjusted. Model 2 is adjusted for sociodemographic variables (sex, age group, race/ethnicity, education, marital status, and living arrangement). Model 3 is adjusted for sociodemographic variables and health insurance type. Model 4 is adjusted for sociodemographic variables, health insurance type, and self-reported severity of symptoms. Models 2–4 are additionally controlled for survey type (online versus phone) and sample.

### Care location

The results of the logistic regression models investigating primary care and ER usage among respondents who sought care are shown in Tables 3 and 4, respectively. Females had higher odds of seeking care from a primary care physician than males (Table 3, Model 2: aOR = 1.72, 95% CI: 1.15, 2.55), while non-Hispanic Black respondents had lower odds of seeking care from a primary care physician (Model 2: aOR = 0.28, 95% CI: 0.17, 0.46) compared to non-Hispanic White respondents. Additionally, respondents who had living arrangements besides owning or renting their homes (which often consisted of staying with a friend or family member) sought care from primary care physicians less often than those who owned their homes (Model 2: aOR = 0.36, 95% CI: 0.18, 0.71). Respondents with a high school education or less had nearly half the odds of seeking care from a primary care physician in comparison to respondents with a college degree (Model 2: aOR = 0.53, 95% CI: 0.31, 0.89). Similarly, the uninsured population had much lower odds of seeking care from a primary care physician compared to respondents with private health insurance (Model 3: aOR = 0.19, 95% CI: 0.09, 0.42). There were no associations between age, marital status, or self-reported symptom severity and seeking care from a primary care physician.

**Table 3:** Predictors of seeking care from a primary care provider using logistic regression (n = 677), MI CReSS

	(1)		(2)		(3)		(4)	
	Odds Ratio	95% CI	Odds Ratio	95% CI	Odds Ratio	95% CI	Odds Ratio	95% CI
Sex								
Male	1.00		1.00		1.00		1.00	
Female	1.34	[0.95, 1.90]	1.72**	[1.15, 2.55]	1.69*	[1.12, 2.54]	1.77**	[1.17, 2.67]
Age Group								
18 to 34	1.00		1.00		1.00		1.00	
35 to 54	1.65	[0.99, 2.75]	1.44	[0.82, 2.53]	1.30	[0.72, 2.38]	1.34	[0.74, 2.42]
55 to 64	1.78*	[1.00, 3.14]	1.52	[0.80, 2.90]	1.44	[0.73, 2.84]	1.50	[0.76, 2.95]
65+	0.88	[0.49, 1.59]	0.78	[0.39, 1.57]	0.71	[0.34, 1.50]	0.69	[0.33, 1.46]
Race/Ethnicity								
Hispanic	0.46**	[0.27, 0.80]	0.65	[0.35, 1.21]	0.87	[0.44, 1.71]	0.92	[0.46, 1.84]
Non-Hispanic White	1.00		1.00		1.00		1.00	
Non-Hispanic Black	0.31***	[0.21, 0.48]	0.28***	[0.17, 0.46]	0.27***	[0.16, 0.45]	0.27***	[0.16, 0.44]
Another race/ethnicity	0.78	[0.45, 1.37]	0.93	[0.50, 1.71]	1.05	[0.55, 2.00]	1.05	[0.56, 2.00]
Annual Household Income								
<$35,000	0.45***	[0.30, 0.68]	0.91	[0.54, 1.54]	1.06	[0.60, 1.86]	1.06	[0.60, 1.87]
$35,000-$74,999	0.50**	[0.32, 0.77]	0.72	[0.44, 1.16]	0.74	[0.45, 1.21]	0.76	[0.46, 1.25]
$75,000+	1.00		1.00		1.00		1.00	
Education								
High school education or less	0.41***	[0.27, 0.63]	0.53*	[0.31, 0.89]	0.52*	[0.31, 0.88]	0.50*	[0.29, 0.85]
Some college or technical school	0.79	[0.52, 1.19]	0.97	[0.61, 1.54]	0.98	[0.61, 1.57]	0.97	[0.60, 1.55]
College graduate	1.00		1.00		1.00		1.00	
Marital Status								
Widowed, divorced, separated, or never married	0.68*	[0.48, 0.97]	1.11	[0.73, 1.70]	1.11	[0.71, 1.71]	1.12	[0.72, 1.73]
Married or living with a partner in a marriage-like relationship	1.00		1.00		1.00		1.00	
Living Arrangement								
Rent	0.49***	[0.32, 0.73]	0.65	[0.40, 1.06]	0.77	[0.46, 1.29]	0.77	[0.46, 1.28]
Own	1.00		1.00		1.00		1.00	
Other arrangement	0.31***	[0.18, 0.56]	0.36**	[0.18, 0.71]	0.44*	[0.22, 0.88]	0.42*	[0.22, 0.83]
Health Insurance Type								
Uninsured	0.17***	[0.08, 0.36]			0.19***	[0.09, 0.42]	0.19***	[0.09, 0.42]
Private	1.00				1.00		1.00	
Medicare	0.40**	[0.23, 0.71]			0.62	[0.31, 1.24]	0.63	[0.31, 1.26]
Medicaid	0.52*	[0.28, 0.98]			0.67	[0.32, 1.41]	0.67	[0.32, 1.41]
Another Type	0.94	[0.32, 2.73]			1.00	[0.31, 3.24]	1.09	[0.34, 3.53]
Self-Reported Severity of Symptoms								
Mild	1.00						1.00	
Moderate	1.24	[0.68, 2.25]					0.66	[0.34, 1.28]
Severe	1.33	[0.76, 2.32]					0.84	[0.44, 1.60]
Very severe	1.01	[0.58, 1.78]					0.68	[0.35, 1.33]

* p < 0.05, ** p < 0.01, *** p < 0.001

This analysis was done using a subsample of respondents who did seek care. The weighted population size was n = 37,499, which reflects the Michigan population with PCR-confirmed COVID-19 who sought care during this period. Model 1 is unadjusted. Model 2 is adjusted for sociodemographic variables (sex, age group, race/ethnicity, education, marital status, and living arrangement). Model 3 is adjusted for sociodemographic variables and health insurance type. Model 4 is adjusted for sociodemographic variables, health insurance type, and self-reported severity of symptoms. Models 2–4 are additionally controlled for survey type (online versus phone) and sample.

**Table 4:** Predictors of seeking care from an emergency room using logistic regression (n = 677), MI CReSS

	(1)		(2)		(3)		(4)	
	Odds Ratio	95% CI	Odds Ratio	95% CI	Odds Ratio	95% CI	Odds Ratio	95% CI
Sex								
Male	1.00		1.00		1.00		1.00	
Female	0.84	[0.59, 1.18]	0.69	[0.47, 1.00]	0.70	[0.47, 1.02]	0.66*	[0.44, 0.99]
Age Group								
18 to 34	1.00		1.00		1.00		1.00	
35 to 54	1.47	[0.88, 2.47]	1.57	[0.87, 2.83]	1.50	[0.83, 2.72]	1.27	[0.70, 2.31]
55 to 64	2.42**	[1.36, 4.28]	2.50**	[1.31, 4.77]	2.39**	[1.25, 4.57]	1.83	[0.94, 3.55]
65+	3.60***	[1.95, 6.67]	3.38***	[1.69, 6.77]	2.93**	[1.42, 6.04]	2.96**	[1.40, 6.28]
Race/Ethnicity								
Hispanic	0.81	[0.47, 1.41]	0.76	[0.41, 1.40]	0.74	[0.39, 1.40]	0.55	[0.29, 1.06]
Non-Hispanic White	1.00		1.00		1.00		1.00	
Non-Hispanic Black	2.01**	[1.32, 3.06]	1.41	[0.87, 2.27]	1.43	[0.88, 2.32]	1.35	[0.82, 2.24]
Another race/ethnicity	0.91	[0.53, 1.56]	0.87	[0.48, 1.55]	0.79	[0.44, 1.44]	0.75	[0.40, 1.41]
Annual Household Income								
<$35,000	2.16***	[1.43, 3.26]	2.03*	[1.18, 3.50]	1.69	[0.95, 3.03]	1.66	[0.90, 3.07]
$35,000-$74,999	1.53*	[1.01, 2.33]	1.52	[0.94, 2.48]	1.51	[0.93, 2.46]	1.34	[0.81, 2.21]
$75,000+	1.00		1.00		1.00		1.00	
Education								
High school education or less	1.65*	[1.08, 2.52]	1.23	[0.74, 2.04]	1.20	[0.72, 2.00]	1.47	[0.84, 2.55]
Some college or technical school	1.79**	[1.19, 2.68]	1.46	[0.95, 2.24]	1.47	[0.95, 2.26]	1.47	[0.95, 2.27]
College graduate	1.00		1.00		1.00		1.00	
Marital Status								
Widowed, divorced, separated, or never married	1.48*	[1.04, 2.10]	1.17	[0.76, 1.80]	1.17	[0.76, 1.79]	1.15	[0.74, 1.79]
Married or living with a partner in a marriage-like relationship	1.00		1.00		1.00		1.00	
Living Arrangement								
Rent	1.38	[0.92, 2.06]	1.30	[0.80, 2.11]	1.20	[0.73, 1.97]	1.20	[0.71, 2.02]
Own	1.00		1.00		1.00		1.00	
Other arrangement	1.71	[0.96, 3.03]	1.94	[0.99, 3.78]	1.72	[0.87, 3.41]	1.92	[0.96, 3.85]
Health Insurance Type								
Uninsured	1.25	[0.66, 2.36]			1.52	[0.77, 2.97]	1.44	[0.72, 2.84]
Private	1.00				1.00		1.00	
Medicare	3.15***	[1.69, 5.86]			2.15*	[1.02, 4.53]	1.99	[0.93, 4.25]
Medicaid	1.26	[0.67, 2.34]			1.43	[0.66, 3.09]	1.37	[0.59, 3.16]
Another Type	1.26	[0.46, 3.49]			1.37	[0.44, 4.20]	1.45	[0.47, 4.52]
Self-Reported Severity of Symptoms								
Mild	1.00						1.00	
Moderate	1.22	[0.65, 2.29]					1.97	[0.99, 3.92]
Severe	2.32**	[1.30, 4.15]					2.90***	[1.55, 5.45]
Very severe	4.95***	[2.73, 8.99]					6.63***	[3.33,13.20]

* p < 0.05, ** p < 0.01, *** p < 0.001

This analysis was done using a subsample of respondents who did seek care. The weighted population size was n = 37,499, which reflects the Michigan population with PCR-confirmed COVID-19 who sought care during this period. Model 1 is unadjusted. Model 2 is adjusted for sociodemographic variables (sex, age group, race/ethnicity, education, marital status, and living arrangement). Model 3 is adjusted for sociodemographic variables and health insurance type. Model 4 is adjusted for sociodemographic variables, health insurance type, and self-reported severity of symptoms. Models 2–4 are additionally controlled for survey type (online versus phone) and sample.

For the analysis of ER utilization, women had 0.69 times the odds of visiting the ER compared to men (Table 4, Model 2: 95% CI: 0.47, 1.00). Increasing age was associated with more ER utilization, such that respondents in the 55- to 64-year-old (Model 2: aOR = 2.50, 95% CI: 1.31, 4.77) and 65+ (Model 2: aOR = 3.38, 95% CI: 1.69, 6.77) age groups had higher odds of visiting the ER for their COVID-19 symptoms than respondents ages 18 to 34. Additionally, individuals who reported an annual income below $35,000 per year (Model 2: aOR = 2.03, 95% CI: 1.18, 3.50) had roughly two times the odds of seeking care for their COVID-19 symptoms in the ER relative to individuals who reported an income over $75,000. Unlike the results seen for primary care, there was an association between self-reported symptom severity and ER utilization. More specifically, respondents with greater severity of symptoms had increasing odds of visiting the ER in comparison to those with mild symptoms (Model 4: Severe: aOR = 2.90, 95% CI: 1.55, 5.45; Very Severe: aOR = 6.63, 95% CI: 3.33, 13.20). There were no associations between race and ethnicity, education, marital status, living arrangement, or health insurance type and seeking care at an ER in the final adjusted models. However, there were statistically significant associations between race and ethnicity, education, marital status, and health insurance type and seeking care at an ER in the unadjusted models.

### Sensitivity analysis

The results of the first sensitivity analysis, examining the predictors of care-seeking for primary care alone, emergency care alone, or both primary care and emergency care can be found in Supplementary Table 1 in Additional File 2. The results of this analysis largely confirm what we found in our main analyses, namely that sex, race and ethnicity, and health insurance were associated with primary care and age group and self-reported symptom severity are associated with emergency care. We also found that age group, race and ethnicity, health insurance, and self-reported severity of symptoms were associated with seeking care both from primary care and the emergency room. We present this as a sensitivity analysis rather than our main analysis due to a low correlation between seeking care at the two locations (R=-0.3409, p < 0.001), and due to small sample sizes in some of the groups.

Additional File 3 contains Supplementary Tables 2–4, the results of the sensitivity analysis adjusting for pre-existing conditions. The results in Supplementary Table 2 (care-seeking analysis) and 3 (primary care analysis) confirmed the results in the main analysis. The results in Supplementary Table 4 (emergency room) differed slightly from the main analysis. More specifically, we found significant associations between identifying as Hispanic, having a household income less than $35,000 per year, having a living arrangement other than owning or renting a home, and seeking care from an ER, respectively, that were not present in the main analysis. However, these associations were all in the same direction as, and of a comparable magnitude to, those in the main analysis, so we do not consider this a cause for concern.

## Discussion

To our knowledge, this is the first study to examine the predictors of care-seeking behaviors in the first months of the COVID-19 pandemic using a population-based study of adults with PCR-confirmed SARS-CoV-2 infection in Michigan. Our results demonstrated that sociodemographic characteristics, health insurance status, and symptom severity were associated with care-seeking behaviors early in the COVID-19 pandemic. The decision to seek care was most influenced by the age of the respondent, as well as their self-reported severity of symptoms. Key predictors varied by the type of facility from which people chose to seek care. Adults who were female, non-Hispanic White, privately insured, lived in high income households, or were highly educated had higher odds of seeking care from a primary care provider compared to their counterparts. Alternatively, adults who were male, older, or had more severe symptoms had higher odds of seeking care from an ER compared to their counterparts. These results indicate differential utilization of healthcare services during the beginning of the pandemic in Michigan and provide the basis for future research on care locations and healthcare access needs during pandemics and other emergency situations.

In our hypothesis, we expected people of lower socioeconomic status, including uninsured individuals and Medicaid recipients, to be less likely to seek care overall, but more likely to visit the ER compared to adults with higher socioeconomic status and private insurance. Assuming people with more severe illness are more likely to seek care from an ER, this would be in line with prior studies which noted that racial and ethnic minoritized populations, as well as those with lower socioeconomic status, were more likely to develop severe COVID-19 illness [30, 31]. In our analysis, seeking care overall, as well as from an ER, was associated with age and self-reported symptom severity, which confirms the prior assumption. However, our results did not show that racial and ethnic minoritized individuals and individuals who had a high school education or less were more likely to seek care overall or from an ER in the adjusted models. A potential explanation for this is that social inequities may have contributed to our results in a different way. Our analyses were only able to include adults who received PCR-testing. Because of the limited availability of COVID-19 testing early in the pandemic, our sample may have been biased towards individuals with more resources and better access to care. Theoretically, this would make it more difficult to detect the results we expected. However, we are unable to ascertain whether social inequities influenced our results in this way without further analyses. Additionally, another limitation of our study was that respondents had to be alive at the time of survey administration, which means that our survey did not capture people who died acutely from COVID-19. Given that racial and ethnic minoritized populations and individuals with lower socioeconomic status were more likely to develop severe COVID-19 illness [30, 31], it follows that these individuals may have been more likely to die before entering our sample. If we examined these associations again during another phase of the pandemic when testing was more readily accessible, or if we were able to better include the population that died from COVID-19, it is possible that our results would be more in line with what we expected. Future pandemic response efforts need to consider these social inequities as they prepare and appropriately allocate resources.

In terms of prior literature on care-seeking behaviors, our results reflected many of the behaviors seen in both day-to-day needs and prior pandemics. For instance, studies investigating age and health insurance in relation to care-seeking behaviors prior to the COVID-19 pandemic reaffirm our findings that older adults are more likely to seek care than their counterparts [4, 7, 10]. Additionally, trends in prior literature indicate that married people are less likely than unmarried people to go to the ER, but more likely to seek care overall [8]. Our results regarding marital status were largely null, and we did not find statistical differences in our adjusted models between married people and unmarried people for seeking care or going to a primary care provider or an ER. Other studies investigating care-seeking trends during prior pandemics, such as the H1N1 pandemic in 2009, also reaffirm the relationships we see in our results. For instance, during the H1N1 pandemic, women and people over the age 65 were more likely to seek care than men or adults ages 18 to 64 [4]. Our results showed that, overall, there were no statistical differences in care-seeking behavior by sex, but women were more likely to visit a primary care provider than men, and men were more likely to go to the ER than women. Additionally, we saw increasing odds of seeking care with age. However, it is important to note that this trend was also seen when looking at ER visits, but not primary care visits. This suggests that the predictors of care-seeking behaviors during the COVID-19 pandemic are comparable to such predictors in prior pandemics.

Despite these similarities, there are also some patterns from prior literature, particularly in day-to-day care-seeking, that were not affirmed in our analyses. For example, prior literature suggests that younger adults are more likely to visit the ER and less likely to visit a primary care provider in comparison to older adults [9]. The opposite patterns came through in our data. Another difference we noticed was that in typical times, Medicaid beneficiaries often use the ER at higher rates than Medicare beneficiaries [12]. In our data, the reverse was true. We saw associations between Medicare and ER usage in our partially adjusted models. However, once we added symptom severity into the models, this association was no longer statistically significant. This is likely because Medicare serves mostly older individuals, and COVID-19 is often more severe in the older population. The fact that these day-to-day care-seeking trends are not reaffirmed in our data may be because the emergence of the COVID-19 pandemic triggered unique care-seeking patterns among certain populations.

Although there have been relatively few studies conducted on care-seeking behaviors during the COVID-19 pandemic, the available research reflects some of the relationships we identified in our analysis. For example, a study of a New York City health system between March 20 and May 18, 2020, found that adults ages 65 + were more likely to seek COVID-19 care from an ER—at least as their first encounter with the healthcare system—rather than through telehealth or at an outpatient office [16]. The same study showed a higher probability of non-Hispanic Black individuals initially seeking care at an ER as opposed to an outpatient office or via telehealth when compared to non-Hispanic White individuals [16]. This relationship was also seen in a study of 13 U.S. states where non-Hispanic Black individuals under the age of 75 were more likely than non-Hispanic White individuals under 75 to seek COVID-19 care from an ER [32]. However, that study reported no difference between non-Hispanic Black individuals and non-Hispanic White individuals when looking at adults over the age of 75 in terms of ER utilization [32]. In our adjusted models, non-Hispanic Black respondents had greater odds of seeking care at an ER compared to non-Hispanic White respondents, but this finding lacked statistical significance. Additionally, our results showed differences by race and ethnicity in seeking care from primary care offices, with non-Hispanic White respondents having higher odds of visiting primary care offices in comparison to non-Hispanic Black respondents. Lastly, a study examining the healthcare utilization of non-hospitalized COVID-19 patients 28–180 days post-diagnosis in a Georgia health system showed several trends similar to our study. A greater percentage of individuals ages 65 + sought care 28–180 days post-diagnosis compared to 18- to 49-year-olds [33]. Likewise, more women sought outpatient care during this timeframe than men [33]. To our knowledge, there have not been any other papers published about the other predictors of seeking COVID-19 care that we investigated in this paper.

Our study design is strong in that it utilizes a population-based sample – something that has not been common in COVID-19 research thus far, but which can provide more generalizable estimates. However, the population comes from a single state, Michigan, during the early stages of the pandemic, so results may not be generalizable to other geographic areas during other timeframes. Additionally, the sampling frame was restricted in a few ways. Firstly, only non-institutionalized adults who were alive at the time of sampling were included. Secondly, the sample was drawn from the MDSS database of individuals with record of a positive COVID-19 PCR test, thus the sample is subject to any biases resulting from limited COVID-19 testing access early in the pandemic. This means that our sample may have a disproportionate number of people who were identified as higher risk patients or worked in healthcare, as these groups were more likely to meet the testing criteria while tests were limited [34]. It also means that our sample may reflect the disproportionate burden of COVID-19 on racial and ethnic minoritized populations, particularly early in the pandemic [14, 35, 36]. The population examined in this study was 52.1% non-Hispanic White, 24.5% non-Hispanic Black, and 11.1% Hispanic respondents (Table 1), while the racial and ethnic breakdown of the Michigan population is 78.8% non-Hispanic White, 14.1% non-Hispanic Black, and 5.7% Hispanic residents [37]. Another potential limitation is non-response bias. We had a response rate of 31.8%, so there is the potential for this bias; however, weights were adjusted to reflect the age and sex distribution of the sampling frame, which helps minimize bias. We also recognize that our survey is subject to reporting bias, as all our variables are self-reported. Respondents may not remember the exact type of facility or may have misclassified one of their care locations. However, since we chose to focus on the two most common types of providers, we believe the probability of misclassification is low in our analysis. An additional important consideration is that our data are cross-sectional and therefore, unable to detect trends in care-seeking over time.

By understanding how people choose to seek care during a pandemic, we can make sure that we are allocating resources to the right places during future public health emergencies. Our results showed that seeking care overall was most strongly associated with age and self-reported severity of symptoms. Similarly, seeking care at an ER was associated with sex, age, and self-reported severity of symptoms. Furthermore, seeking care from a primary care provider was associated with sex, race and ethnicity, living arrangement, education, and type of health insurance. This evidence provides the foundation for future research needed on care-seeking behaviors. Additional studies examining specific barriers to seeking care in the COVID-19 pandemic may help elucidate necessary interventions. More specifically, investigation of communication failures, paid sick leave, the availability of childcare services, and access to transportation would provide further insight into unmet needs during the COVID-19 pandemic, and potential inequities that could be resolved through policy intervention.

## Conclusion

Our analysis demonstrated that age and self-reported severity of symptoms were associated with whether adult Michiganders sought care for COVID-19. Additionally, sex, race and ethnicity, living arrangement, education, and type of health insurance were associated with seeking care from a primary care physician. In contrast, sex, age, and self-reported severity of symptoms were associated with seeking care from an ER. Future efforts should focus on promoting access to care and breaking down barriers to receiving care in order to achieve health equity.

### Electronic supplementary material

Below is the link to the electronic supplementary material.

Supplementary Material 1

Supplementary Material 2

Supplementary Material 3

## Data Availability

Although the dataset used in this study is not currently available to others, we are in the process of making a de-identified dataset and data dictionary available. Any requests for the data in the interim can be sent to the MI CReSS study team at michigan-cress@umich.edu.

## Abbreviations

ER: Emergency room
MI CReSS: Michigan COVID-19 Recovery Surveillance Study
MDSS: Michigan Disease Surveillance System
PCP: Primary care physician/family doctor

## References

[1] Czeisler ME, Marynak K, Clarke KEN, Salah Z, Shakya I, Thierry JM (2020). Delay or Avoidance of Medical Care because of COVID-19-Related concerns - United States, June 2020. MMWR Morb Mortal Wkly Rep.

[2] Hartnett KP, Kite-Powell A, DeVies J, Coletta MA, Boehmer TK, Adjemian J (2020). Impact of the COVID-19 pandemic on Emergency Department visits - United States, January 1, 2019-May 30, 2020. MMWR Morb Mortal Wkly Rep.

[3] Mehrotra A, Chernew M, Linetsky D, Hatch H, Cutler D, Schneider E. The impact of the COVID-19 pandemic on outpatient visits: changing patterns of care in the newest COVID-19 hot spots. The Commonwealth Fund2020 August 13, 2020.

[4] Biggerstaff M, Jhung MA, Reed C, Garg S, Balluz L, Fry AM (2014). Impact of medical and behavioural factors on influenza-like Illness, healthcare-seeking, and antiviral treatment during the 2009 H1N1 pandemic: USA, 2009–2010. Epidemiol Infect.

[5] Baltrusaitis K, Reed C, Sewalk K, Brownstein JS, Crawley AW, Biggerstaff M (2022). Healthcare-seeking behavior for respiratory Illness among Flu Near you participants in the United States during the 2015–2016 through 2018–2019 Influenza Seasons. J Infect Dis.

[6] Ma W, Huo X, Zhou M (2018). The healthcare seeking rate of individuals with Influenza like Illness: a meta-analysis. Infect Dis (Lond).

[7] Biggerstaff M, Jhung MA, Reed C, Fry AM, Balluz L, Finelli L (2014). Influenza-like Illness, the time to seek healthcare, and Influenza antiviral receipt during the 2010–2011 Influenza season-United States. J Infect Dis.

[8] Zheng X, Zimmer D (2009). Racial differences in Health-care utilization: analysis by intensity of demand. Contemp Econ Policy.

[9] Fortuna RJ, Robbins BW, Mani N, Halterman JS (2010). Dependence on emergency care among young adults in the United States. J Gen Intern Med.

[10] Institute of Medicine (U.S.) (2008). Committee on the Future Health Care workforce for older americans. Retooling for an aging America: building the health care workforce.

[11] Brown LE, Burton R, Hixon B, Kakade M, Bhagalia P, Vick C (2012). Factors influencing emergency department preference for access to healthcare. West J Emerg Med.

[12] Cairns C, Ashman JJ, Kang K (2021). Emergency Department visit rates by selected characteristics: United States, 2018. NCHS Data Brief.

[13] Kangovi S, Barg FK, Carter T, Long JA, Shannon R, Grande D (2013). Understanding why patients of low socioeconomic status prefer hospitals over ambulatory care. Health Aff (Millwood).

[14] Michigan Data. : Michigan Department of Health and Human Services2020.

[15] Levitan EB, Howard VJ, Cushman M, Judd SE, Tison SE, Yuan Y (2021). Health care experiences during the COVID-19 pandemic by race and social determinants of health among adults age >/= 58 years in the REGARDS study. BMC Public Health.

[16] Weber E, Miller SJ, Astha V, Janevic T, Benn E (2020). Characteristics of telehealth users in NYC for COVID-related care during the coronavirus pandemic. J Am Med Inform Assoc.

[17] Colchero MA, Moreno-Aguilar LA, Bautista-Arredondo SA. The Covid-19 cascade of care in Mexico: symptoms, positivity, and health care seeking decisions amid the pandemic. Salud Publica Mex. 2021;63(6, Nov-Dic):734 – 42. 10.21149/12822 35099913

[18] Yang J, Gong H, Chen X, Chen Z, Deng X, Qian M (2021). Health-seeking behaviors of patients with acute Respiratory Infections during the outbreak of novel coronavirus Disease 2019 in Wuhan, China. Influenza Other Respir Viruses.

[19] Tay MZ, Ang LW, Wei WE, Lee VJM, Leo YS, Toh M (2022). Health-seeking behavior of COVID-19 cases during the first eight weeks of the outbreak in Singapore: differences between local community and imported cases and having visits to single or multiple healthcare providers. BMC Public Health.

[20] Nunez A, Sreeganga SD, Ramaprasad A. Access to Healthcare during COVID-19. Int J Environ Res Public Health. 2021;18(6). 10.3390/ijerph18062980 PMC7999346 33799417

[21] Michigan COVID-. 19 Recovery Surveillance Study. 2020.

[22] Standard definitions. Final dispositions of case codes and outcome rates for surveys. 9th ed. American Association for Public Opinion Research; 2016.

[23] Deville J, Sarndal C (1992). Calibration estimators in survey sampling. J Am Stat Assoc.

[24] Cox B. The weighted sequential hot deck imputation procedure. ASA Proc Section on Survey Res Methods. 1980:721–6.

[25] Mayer B (2013). Hot deck propensity score imputation for missing values. Sci J Med Clin Trials.

[26] How Medicare works with other insurance. Medicare.gov [cited 2021 January 31, 2021]; Available from: https://www.medicare.gov/supplements-other-insurance/how-medicare-works-with-other-insurance.

[27] Third Party Liability. : What is a Third Party? Michigan Department of Health and Human Services; [cited 2021 January 31, 2021]; Available from: https://www.michigan.gov/mdhhs/0,5885,7-339-71547_4860-240787--,00.html.

[28] Inc SI (2016). SAS Software 9.4. Cary.

[29] StataCorp (2021). Stata Statistical Software: Release 17. College Station.

[30] Clay SL, Woodson MJ, Mazurek K, Antonio B (2021). Racial disparities and COVID-19: exploring the relationship between Race/Ethnicity, personal factors, Health Access/Affordability, and conditions Associated with an increased severity of COVID-19. Race Soc Probl.

[31] Hawkins RB, Charles EJ, Mehaffey JH (2020). Socio-economic status and COVID-19-related cases and fatalities. Public Health.

[32] Smith AR, DeVies J, Caruso E, Radhakrishnan L, Sheppard M, Stein Z (2021). Emergency Department visits for COVID-19 by race and ethnicity – 13 States, October-December 2020. MMWR Morb Mortal Wkly Rep.

[33] Hernandez-Romieu AC, Leung S, Mbanya A, Jackson BR, Cope JR, Bushman D (2021). Health Care utilization and clinical characteristics of nonhospitalized adults in an Integrated Health Care System 28–180 days after COVID-19 diagnosis - Georgia, May 2020-March 2021. MMWR Morb Mortal Wkly Rep.

[34] Updated Guidance on Evaluating and Testing Persons for Coronavirus Disease 2019 (COVID-19). Centers for Disease Control and Prevention; 2020 [cited 2023 July 27]; Available from: https://emergency.cdc.gov/han/2020/han00429.asp.

[35] Bibbins-Domingo K (2020). This time must be different: disparities during the COVID-19 pandemic. Ann Intern Med.

[36] Gu T, Mack JA, Salvatore M, Prabhu Sankar S, Valley TS, Singh K (2020). Characteristics Associated with Racial/Ethnic disparities in COVID-19 outcomes in an Academic Health Care System. JAMA Netw Open.

[37] QuickFacts M. United States Census Bureau; [cited 2023 July 14]; Available from: https://www.census.gov/quickfacts/MI.

