# Supplementary material for: Differential care-seeking behaviors during the beginning of the COVID-19 pandemic in Michigan: a population-based cross-sectional study
Source: BMC Public Health. 2023 Oct 25;23:2101. doi: 10.1186/s12889-023-16999-5 (PMC10601223; doi:10.1186/s12889-023-16999-5)
Supplement: Supplementary file 1 — Supplementary Material 1 [file 12889_2023_16999_MOESM1_ESM.pptx]

## Slide 1
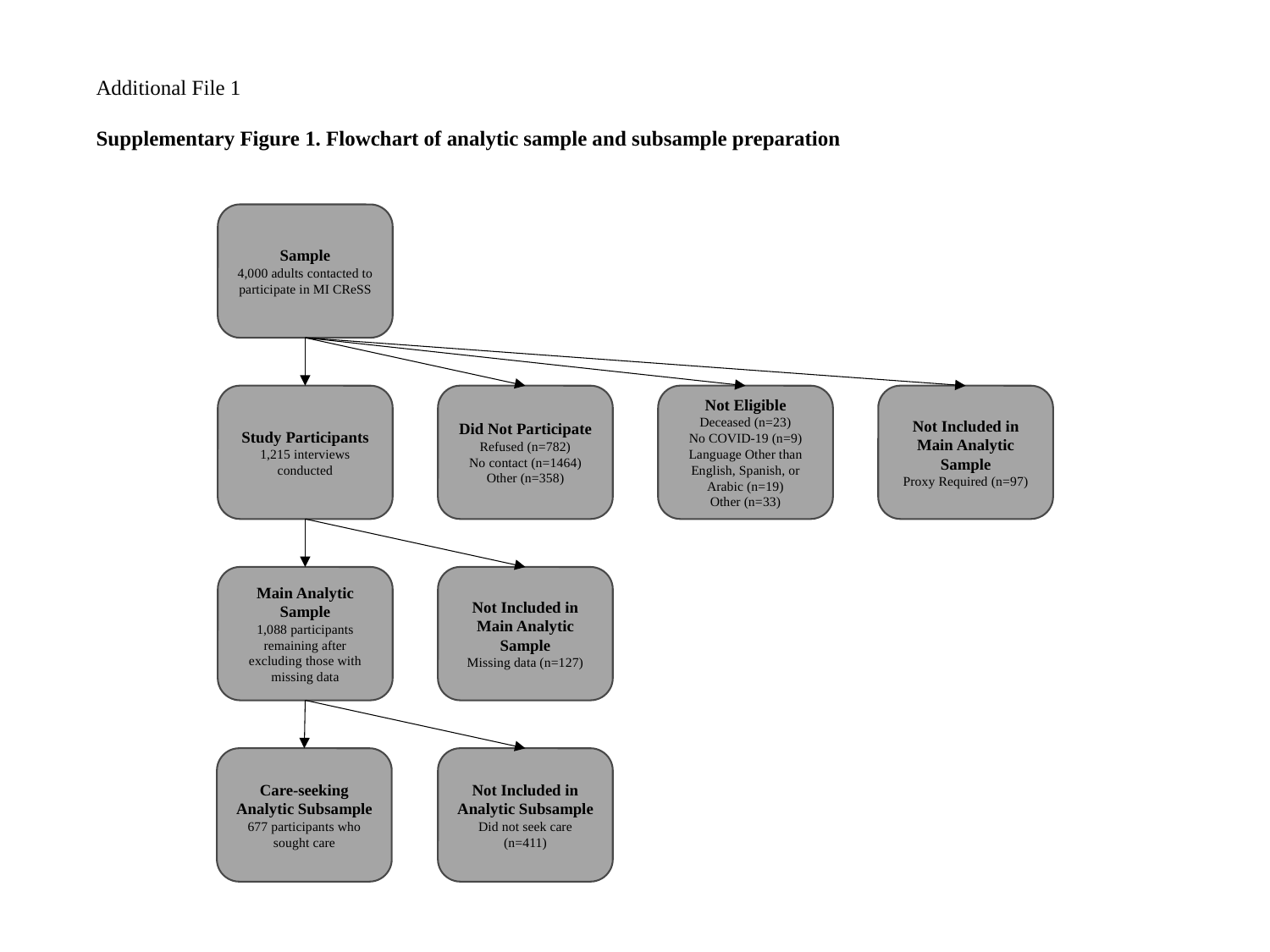

Additional File 1
Supplementary Figure 1. Flowchart of analytic sample and subsample preparation
Sample
4,000 adults contacted to participate in MI CReSS
Not Included in Main Analytic Sample
Proxy Required (n=97)
Not Eligible
Deceased (n=23)
No COVID-19 (n=9)
Language Other than English, Spanish, or Arabic (n=19)
Other (n=33)
Study Participants
1,215 interviews conducted
Did Not Participate
Refused (n=782)
No contact (n=1464)
Other (n=358)
Main Analytic Sample
1,088 participants remaining after excluding those with missing data
Not Included in Main Analytic Sample
Missing data (n=127)
Care-seeking Analytic Subsample
677 participants who sought care
Not Included in Analytic Subsample
Did not seek care (n=411)
