# Supplementary material for: Differential care-seeking behaviors during the beginning of the COVID-19 pandemic in Michigan: a population-based cross-sectional study
Source: BMC Public Health. 2023 Oct 25;23:2101. doi: 10.1186/s12889-023-16999-5 (PMC10601223; doi:10.1186/s12889-023-16999-5)
Supplement: Supplementary file 2 — Supplementary Material 2 [file 12889_2023_16999_MOESM2_ESM.docx]

Additional File 2

| **Supplementary Table 1. Sensitivity analysis using an alternate outcome definition: Predictors of seeking care from a primary care physician, emergency room, or neither among those who sought care using multinomial logistic regression (n=677), Michigan COVID-19 Recovery Surveillance Study** | | | | | | | |
| --- | --- | --- | --- | --- | --- | --- | --- |
|  | Sought care from a PCP, but not the ER (n=249) | | Sought care from the ER, but not a PCP (n=197) | | Sought care from a PCP and the ER (n=150) | | Sought care, but not from a PCP or the ER  (reference group) (n=81) |
|  | Relative Risk Ratio | 95% CI | Relative Risk Ratio | 95% CI | Relative Risk Ratio | 95% CI | Relative Risk Ratio |
| Sex |  |  |  |  |  |  |  |
| Male | 1.00 |  | 1.00 |  | 1.00 |  | 1.00 |
| Female | 2.50** | [1.32, 4.76] | 1.25 | [0.64, 2.44] | 1.45 | [0.71, 2.95] | 1.00 |
|  |  |  |  |  |  |  |  |
| Age Group | |  |  |  |  |  |  |
| 18 to 34 | 1.00 |  | 1.00 |  | 1.00 |  | 1.00 |
| 35 to 54 | 1.19 | [0.52, 2.72] | 1.11 | [0.46, 2.69] | 2.02 | [0.71, 5.71] | 1.00 |
| 55 to 64 | 2.73 | [0.89, 8.37] | 3.13* | [1.00, 9.76] | 5.95** | [1.68,21.10] | 1.00 |
| 65+ | 0.94 | [0.31, 2.88] | 2.71 | [0.90, 8.17] | 2.96 | [0.86,10.20] | 1.00 |
|  |  |  |  |  |  |  |  |
| Race and Ethnicity | |  |  |  |  |  |  |
| Hispanic | 1.08 | [0.38, 3.05] | 0.76 | [0.27, 2.14] | 0.39 | [0.12, 1.25] | 1.00 |
| Non-Hispanic White | 1.00 |  | 1.00 |  | 1.00 |  | 1.00 |
| Non-Hispanic Black | 0.40* | [0.17, 0.94] | 1.30 | [0.58, 2.93] | 0.24** | [0.10, 0.58] | 1.00 |
| Another race/ethnicity | 1.10 | [0.40, 3.06] | 0.87 | [0.28, 2.67] | 0.75 | [0.23, 2.50] | 1.00 |
|  |  |  |  |  |  |  |  |
| Annual Household Income | | |  |  |  |  |  |
| <$35,000 | 1.09 | [0.42, 2.85] | 1.50 | [0.52, 4.36] | 2.39 | [0.79, 7.19] | 1.00 |
| $35,000-$74,999 | 0.57 | [0.26, 1.27] | 0.83 | [0.34, 1.99] | 0.92 | [0.38, 2.27] | 1.00 |
| $75,000+ | 1.00 |  | 1.00 |  | 1.00 |  | 1.00 |
|  |  |  |  |  |  |  |  |
| Education |  |  |  |  |  |  |  |
| High school education or less | 0.53 | [0.23, 1.21] | 1.20 | [0.51, 2.82] | 0.57 | [0.23, 1.45] | 1.00 |
| Some college or technical school | 1.71 | [0.79, 3.69] | 2.38* | [1.07, 5.26] | 1.96 | [0.85, 4.50] | 1.00 |
| College graduate | 1.00 |  | 1.00 |  | 1.00 |  | 1.00 |
|  |  |  |  |  |  |  |  |
| Marital Status | |  |  |  |  |  |  |
| Widowed, divorced, separated, or never married | 0.82 | [0.38, 1.76] | 0.81 | [0.38, 1.71] | 1.33 | [0.59, 3.01] | 1.00 |
| Married or living with a partner in a marriage-like relationship | 1.00 |  | 1.00 |  | 1.00 |  | 1.00 |
|  |  |  |  |  |  |  |  |
| Living Arrangement | |  |  |  |  |  |  |
| Rent | 0.80 | [0.36, 1.75] | 1.09 | [0.47, 2.52] | 0.95 | [0.40, 2.26] | 1.00 |
| Own | 1.00 |  | 1.00 |  | 1.00 |  | 1.00 |
| Other arrangement | 0.74 | [0.25, 2.20] | 2.16 | [0.77, 6.08] | 0.78 | [0.23, 2.67] | 1.00 |
|  |  |  |  |  |  |  |  |
| Health Insurance | |  |  |  |  |  |  |
| Uninsured | 0.13*** | [0.04, 0.38] | 0.60 | [0.23, 1.56] | 0.13** | [0.03, 0.51] | 1.00 |
| Private | 1.00 |  | 1.00 |  | 1.00 |  | 1.00 |
| Medicare | 0.78 | [0.21, 2.96] | 1.84 | [0.49, 6.88] | 1.30 | [0.32, 5.25] | 1.00 |
| Medicaid | 0.56 | [0.17, 1.81] | 0.94 | [0.28, 3.22] | 0.70 | [0.18, 2.72] | 1.00 |
| Another Type | 0.98 | [0.17, 5.84] | 1.17 | [0.15, 9.03] | 1.75 | [0.20,15.58] | 1.00 |
|  |  |  |  |  |  |  |  |
| Self-Reported Symptom Severity | | | |  |  |  |  |
| Mild | 1.00 |  | 1.00 |  | 1.00 |  | 1.00 |
| Moderate | 0.94 | [0.37, 2.38] | 2.26 | [0.80, 6.41] | 1.42 | [0.41, 4.86] | 1.00 |
| Severe | 1.03 | [0.43, 2.48] | 2.58* | [1.00, 6.62] | 3.51* | [1.20,10.33] | 1.00 |
| Very severe | 1.86 | [0.61, 5.65] | 9.97*** | [3.27,30.35] | 12.22*** | [3.45,43.28] | 1.00 |
| * p<0.05, ** p<0.01, *** p<0.001 | | | |  |  |  |  |
| This model is adjusted for sociodemographic variables (sex, age group, race/ethnicity, education, marital status, and living arrangement), health insurance type, self-reported severity of symptoms, survey type (online versus phone), and sample. It is analogous to the fully adjusted models (Model 4) in the main paper. | | | | | | | |
