# Supplementary material for: Differential care-seeking behaviors during the beginning of the COVID-19 pandemic in Michigan: a population-based cross-sectional study
Source: BMC Public Health. 2023 Oct 25;23:2101. doi: 10.1186/s12889-023-16999-5 (PMC10601223; doi:10.1186/s12889-023-16999-5)
Supplement: Supplementary file 3 — Supplementary Material 3 [file 12889_2023_16999_MOESM3_ESM.docx]

Additional File 3

| **Supplementary Table 2. Sensitivity analysis controlling for comorbidities: Predictors of seeking care for COVID-19 versus not seeking care using logistic regression (n=1020), Michigan COVID-19 Recovery Surveillance Study** | | | | | | | | |
| --- | --- | --- | --- | --- | --- | --- | --- | --- |
|  | (1) | | (2) | | (3) | | (4) | |
|  | Odds Ratio | 95% CI | Odds Ratio | 95% CI | Odds Ratio | 95% CI | Odds Ratio | 95% CI |
| Sex |  |  |  |  |  |  |  |  |
| Male | 1.00 |  | 1.00 |  | 1.00 |  | 1.00 |  |
| Female | 1.04 | [0.78, 1.40] | 1.06 | [0.77, 1.47] | 1.08 | [0.78, 1.49] | 1.01 | [0.72, 1.43] |
|  |  |  |  |  |  |  |  |  |
| Age Group |  |  |  |  |  |  |  |  |
| 18 to 34 | 1.00 |  | 1.00 |  | 1.00 |  | 1.00 |  |
| 35 to 54 | 3.65*** | [2.52, 5.28] | 2.85*** | [1.87, 4.36] | 2.91*** | [1.90, 4.46] | 2.66*** | [1.71, 4.15] |
| 55 to 64 | 3.86*** | [2.48, 6.01] | 2.46*** | [1.46, 4.16] | 2.51*** | [1.49, 4.25] | 2.05* | [1.17, 3.59] |
| 65+ | 4.81*** | [2.95, 7.84] | 2.63** | [1.43, 4.82] | 2.69** | [1.44, 5.02] | 3.01*** | [1.56, 5.78] |
|  |  |  |  |  |  |  |  |  |
| Race/Ethnicity |  |  |  |  |  |  |  |  |
| Hispanic | 1.65 | [1.00, 2.74] | 1.61 | [0.89, 2.92] | 1.52 | [0.83, 2.76] | 1.11 | [0.57, 2.15] |
| Non-Hispanic White | 1.00 |  | 1.00 |  | 1.00 |  | 1.00 |  |
| Non-Hispanic Black | 1.73** | [1.19, 2.50] | 1.03 | [0.68, 1.58] | 1.04 | [0.68, 1.59] | 0.96 | [0.61, 1.51] |
| Another race/ethnicity | 1.56 | [0.98, 2.49] | 1.56 | [0.92, 2.65] | 1.54 | [0.90, 2.62] | 1.44 | [0.82, 2.52] |
|  |  |  |  |  |  |  |  |  |
| Annual Household Income |  |  |  |  |  |  |  |  |
| <$35,000 | 1.39 | [0.98, 1.96] | 1.34 | [0.83, 2.15] | 1.28 | [0.77, 2.12] | 1.26 | [0.73, 2.19] |
| $35,000-$74,999 | 1.15 | [0.81, 1.62] | 1.19 | [0.80, 1.77] | 1.16 | [0.78, 1.74] | 1.02 | [0.66, 1.58] |
| $75,000+ | 1.00 |  | 1.00 |  | 1.00 |  | 1.00 |  |
|  |  |  |  |  |  |  |  |  |
| Education |  |  |  |  |  |  |  |  |
| High school education or less | 1.63** | [1.13, 2.36] | 1.06 | [0.68, 1.67] | 1.05 | [0.67, 1.65] | 1.32 | [0.82, 2.14] |
| Some college or technical school | 1.25 | [0.90, 1.74] | 1.13 | [0.78, 1.64] | 1.12 | [0.77, 1.62] | 1.12 | [0.76, 1.66] |
| College graduate | 1.00 |  | 1.00 |  | 1.00 |  | 1.00 |  |
|  |  |  |  |  |  |  |  |  |
| Marital Status |  |  |  |  |  |  |  |  |
| Widowed, divorced, separated, or never married | 0.75 | [0.56, 1.01] | 0.88 | [0.61, 1.28] | 0.88 | [0.60, 1.28] | 0.87 | [0.58, 1.31] |
| Married or living with a partner in a marriage-like relationship | 1.00 |  | 1.00 |  | 1.00 |  | 1.00 |  |
|  |  |  |  |  |  |  |  |  |
| Living Arrangement |  |  |  |  |  |  |  |  |
| Rent | 0.79 | [0.56, 1.10] | 0.99 | [0.64, 1.53] | 0.96 | [0.61, 1.49] | 0.87 | [0.55, 1.39] |
| Own | 1.00 |  | 1.00 |  | 1.00 |  | 1.00 |  |
| Other arrangement | 0.70 | [0.45, 1.08] | 1.10 | [0.63, 1.92] | 1.06 | [0.60, 1.87] | 1.02 | [0.55, 1.89] |
|  |  |  |  |  |  |  |  |  |
| Comorbidity Index |  |  |  |  |  |  |  |  |
| 0 Comorbidities | 1.00 |  | 1.00 |  | 1.00 |  | 1.00 |  |
| 1 Comorbidity | 1.61* | [1.12, 2.31] | 1.35 | [0.90, 2.03] | 1.37 | [0.91, 2.06] | 1.23 | [0.80, 1.89] |
| 2 Comorbidities | 2.60*** | [1.70, 3.98] | 1.78* | [1.12, 2.82] | 1.81* | [1.13, 2.88] | 1.50 | [0.92, 2.45] |
| ≥3 Comorbidities | 3.33*** | [2.19, 5.07] | 1.86* | [1.14, 3.03] | 1.90* | [1.16, 3.11] | 1.54 | [0.92, 2.56] |
|  |  |  |  |  |  |  |  |  |
| Health Insurance Type |  |  |  |  |  |  |  |  |
| Uninsured | 1.07 | [0.64, 1.79] |  |  | 1.59 | [0.87, 2.89] | 1.57 | [0.83, 2.97] |
| Private | 1.00 |  |  |  | 1.00 |  | 1.00 |  |
| Medicare | 1.74 | [0.98, 3.09] |  |  | 1.08 | [0.54, 2.15] | 1.01 | [0.49, 2.09] |
| Medicaid | 1.13 | [0.66, 1.93] |  |  | 1.06 | [0.53, 2.12] | 1.08 | [0.48, 2.43] |
| Another Type | 1.17 | [0.49, 2.82] |  |  | 1.01 | [0.39, 2.61] | 1.03 | [0.40, 2.67] |
|  |  |  |  |  |  |  |  |  |
| Self-Reported Severity of Symptoms |  |  |  |  |  |  |  |  |
| Mild | 1.00 |  |  |  |  |  | 1.00 |  |
| Moderate | 2.32*** | [1.54, 3.48] |  |  |  |  | 2.20*** | [1.42, 3.41] |
| Severe | 4.19*** | [2.80, 6.27] |  |  |  |  | 3.28*** | [2.13, 5.05] |
| Very severe | 19.91*** | [10.63,37.31] |  |  |  |  | 13.73*** | [7.08,26.60] |
| * p<0.05, ** p<0.01, *** p<0.001 Model 1 is unadjusted. Model 2 is adjusted for sociodemographic variables (sex, age group, race/ethnicity, education, marital status, and living arrangement). Model 3 is adjusted for sociodemographic variables and health insurance type. Model 4 is adjusted for sociodemographic variables, health insurance type, and self-reported severity of symptoms. Models 2-4 are additionally controlled for survey type (online versus phone) and sample. | | | | | | | | |

| **Supplementary Table 3. Sensitivity analysis controlling for comorbidities: Predictors of seeking care from a primary care physician or family doctor versus somewhere else among those who sought care using logistic regression (n=639), Michigan COVID-19 Recovery Surveillance Study** | | | | | | | | |
| --- | --- | --- | --- | --- | --- | --- | --- | --- |
|  | (1) | | (2) | | (3) | | (4) | |
|  | Odds Ratio | 95% CI | Odds Ratio | 95% CI | Odds Ratio | 95% CI | Odds Ratio | 95% CI |
| Sex |  |  |  |  |  |  |  |  |
| Male | 1.00 |  | 1.00 |  | 1.00 |  | 1.00 |  |
| Female | 1.29 | [0.90, 1.85] | 1.61* | [1.07, 2.40] | 1.54* | [1.02, 2.34] | 1.62* | [1.06, 2.47] |
|  |  |  |  |  |  |  |  |  |
| Age Group |  |  |  |  |  |  |  |  |
| 18 to 34 | 1.00 |  | 1.00 |  | 1.00 |  | 1.00 |  |
| 35 to 54 | 1.59 | [0.95, 2.69] | 1.35 | [0.73, 2.48] | 1.22 | [0.64, 2.31] | 1.27 | [0.67, 2.39] |
| 55 to 64 | 1.72 | [0.96, 3.09] | 1.42 | [0.71, 2.83] | 1.36 | [0.66, 2.78] | 1.44 | [0.70, 2.96] |
| 65+ | 0.82 | [0.45, 1.49] | 0.67 | [0.31, 1.46] | 0.64 | [0.28, 1.45] | 0.63 | [0.28, 1.41] |
|  |  |  |  |  |  |  |  |  |
| Race/Ethnicity |  |  |  |  |  |  |  |  |
| Hispanic | 0.47** | [0.26, 0.82] | 0.66 | [0.35, 1.27] | 0.93 | [0.45, 1.92] | 1.01 | [0.49, 2.10] |
| Non-Hispanic White | 1.00 |  | 1.00 |  | 1.00 |  | 1.00 |  |
| Non-Hispanic Black | 0.30*** | [0.20, 0.47] | 0.27*** | [0.16, 0.46] | 0.26*** | [0.16, 0.44] | 0.26*** | [0.15, 0.44] |
| Another race/ethnicity | 0.82 | [0.46, 1.46] | 0.99 | [0.52, 1.88] | 1.13 | [0.58, 2.22] | 1.14 | [0.59, 2.22] |
|  |  |  |  |  |  |  |  |  |
| Annual Household Income |  |  |  |  |  |  |  |  |
| <$35,000 | 0.40*** | [0.26, 0.61] | 0.78 | [0.45, 1.38] | 0.88 | [0.48, 1.63] | 0.89 | [0.48, 1.64] |
| $35,000-$74,999 | 0.48** | [0.31, 0.75] | 0.68 | [0.41, 1.13] | 0.71 | [0.42, 1.20] | 0.74 | [0.43, 1.25] |
| $75,000+ | 1.00 |  | 1.00 |  | 1.00 |  | 1.00 |  |
|  |  |  |  |  |  |  |  |  |
| Education |  |  |  |  |  |  |  |  |
| High school education or less | 0.39*** | [0.25, 0.61] | 0.50* | [0.29, 0.88] | 0.49* | [0.28, 0.86] | 0.45** | [0.25, 0.80] |
| Some college or technical school | 0.73 | [0.48, 1.12] | 0.92 | [0.57, 1.50] | 0.94 | [0.57, 1.53] | 0.91 | [0.56, 1.49] |
| College graduate | 1.00 |  | 1.00 |  | 1.00 |  | 1.00 |  |
|  |  |  |  |  |  |  |  |  |
| Marital Status |  |  |  |  |  |  |  |  |
| Widowed, divorced, separated, or never married | 0.64* | [0.44, 0.92] | 1.07 | [0.69, 1.67] | 1.08 | [0.68, 1.71] | 1.09 | [0.69, 1.74] |
| Married or living with a partner in a marriage-like relationship | 1.00 |  | 1.00 |  | 1.00 |  | 1.00 |  |
|  |  |  |  |  |  |  |  |  |
| Living Arrangement |  |  |  |  |  |  |  |  |
| Rent | 0.51** | [0.34, 0.78] | 0.73 | [0.44, 1.22] | 0.85 | [0.50, 1.45] | 0.85 | [0.50, 1.46] |
| Own | 1.00 |  | 1.00 |  | 1.00 |  | 1.00 |  |
| Other arrangement | 0.27*** | [0.15, 0.50] | 0.32** | [0.15, 0.67] | 0.39* | [0.19, 0.82] | 0.37** | [0.18, 0.76] |
|  |  |  |  |  |  |  |  |  |
| Comorbidity Index |  |  |  |  |  |  |  |  |
| 0 Comorbidities | 1.00 |  | 1.00 |  | 1.00 |  | 1.00 |  |
| 1 Comorbidity | 0.80 | [0.49, 1.31] | 0.75 | [0.44, 1.28] | 0.72 | [0.42, 1.26] | 0.74 | [0.42, 1.28] |
| 2 Comorbidities | 0.98 | [0.59, 1.63] | 1.01 | [0.56, 1.80] | 1.00 | [0.55, 1.85] | 1.05 | [0.57, 1.94] |
| ≥3 Comorbidities | 0.80 | [0.49, 1.30] | 0.97 | [0.55, 1.73] | 0.95 | [0.52, 1.73] | 0.99 | [0.54, 1.80] |
|  |  |  |  |  |  |  |  |  |
| Health Insurance Type |  |  |  |  |  |  |  |  |
| Uninsured | 0.16*** | [0.07, 0.35] |  |  | 0.18*** | [0.08, 0.40] | 0.18*** | [0.08, 0.39] |
| Private | 1.00 |  |  |  | 1.00 |  | 1.00 |  |
| Medicare | 0.38** | [0.21, 0.69] |  |  | 0.57 | [0.27, 1.20] | 0.58 | [0.27, 1.21] |
| Medicaid | 0.57 | [0.30, 1.09] |  |  | 0.78 | [0.36, 1.67] | 0.79 | [0.37, 1.69] |
| Another Type | 1.16 | [0.37, 3.61] |  |  | 1.52 | [0.49, 4.64] | 1.64 | [0.53, 5.11] |
|  |  |  |  |  |  |  |  |  |
| Self-Reported Severity of Symptoms |  |  |  |  |  |  |  |  |
| Mild | 1.00 |  |  |  |  |  | 1.00 |  |
| Moderate | 1.29 | [0.70, 2.38] |  |  |  |  | 0.60 | [0.29, 1.22] |
| Severe | 1.30 | [0.73, 2.31] |  |  |  |  | 0.71 | [0.36, 1.41] |
| Very severe | 0.96 | [0.54, 1.72] |  |  |  |  | 0.57 | [0.28, 1.15] |
| * p<0.05, ** p<0.01, *** p<0.001 Model 1 is unadjusted. Model 2 is adjusted for sociodemographic variables (sex, age group, race/ethnicity, education, marital status, and living arrangement). Model 3 is adjusted for sociodemographic variables and health insurance type. Model 4 is adjusted for sociodemographic variables, health insurance type, and self-reported severity of symptoms. Models 2-4 are additionally controlled for survey type (online versus phone) and sample. | | | | | | | | |

| **Supplementary Table 4. Sensitivity analysis controlling for comorbidities: Predictors of seeking care from an emergency room versus somewhere else among those who sought care using logistic regression (n=639), Michigan COVID-19 Recovery Surveillance Study** | | | | | | | | |
| --- | --- | --- | --- | --- | --- | --- | --- | --- |
|  | (1) | | (2) | | (3) | | (4) | |
|  | Odds Ratio | 95% CI | Odds Ratio | 95% CI | Odds Ratio | 95% CI | Odds Ratio | 95% CI |
| Sex |  |  |  |  |  |  |  |  |
| Male | 1.00 |  | 1.00 |  | 1.00 |  | 1.00 |  |
| Female | 0.90 | [0.63, 1.28] | 0.75 | [0.51, 1.11] | 0.77 | [0.52, 1.14] | 0.73 | [0.48, 1.11] |
|  |  |  |  |  |  |  |  |  |
| Age Group |  |  |  |  |  |  |  |  |
| 18 to 34 | 1.00 |  | 1.00 |  | 1.00 |  | 1.00 |  |
| 35 to 54 | 1.33 | [0.78, 2.25] | 1.37 | [0.73, 2.54] | 1.34 | [0.72, 2.51] | 1.14 | [0.61, 2.16] |
| 55 to 64 | 2.35** | [1.31, 4.21] | 2.17* | [1.07, 4.40] | 2.12* | [1.04, 4.31] | 1.66 | [0.79, 3.50] |
| 65+ | 3.17*** | [1.69, 5.98] | 2.55* | [1.18, 5.54] | 2.32* | [1.05, 5.14] | 2.41* | [1.05, 5.52] |
|  |  |  |  |  |  |  |  |  |
| Race/Ethnicity |  |  |  |  |  |  |  |  |
| Hispanic | 0.78 | [0.44, 1.37] | 0.65 | [0.34, 1.25] | 0.61 | [0.31, 1.21] | 0.44* | [0.22, 0.89] |
| Non-Hispanic White | 1.00 |  | 1.00 |  | 1.00 |  | 1.00 |  |
| Non-Hispanic Black | 2.04** | [1.32, 3.14] | 1.36 | [0.83, 2.24] | 1.38 | [0.84, 2.28] | 1.35 | [0.80, 2.27] |
| Another race/ethnicity | 0.87 | [0.50, 1.52] | 0.76 | [0.42, 1.39] | 0.72 | [0.39, 1.32] | 0.65 | [0.34, 1.25] |
|  |  |  |  |  |  |  |  |  |
| Annual Household Income |  |  |  |  |  |  |  |  |
| <$35,000 | 2.47*** | [1.61, 3.78] | 2.29** | [1.28, 4.08] | 2.02* | [1.08, 3.80] | 1.99* | [1.02, 3.90] |
| $35,000-$74,999 | 1.67* | [1.09, 2.57] | 1.65 | [1.00, 2.71] | 1.62 | [0.98, 2.67] | 1.42 | [0.84, 2.40] |
| $75,000+ | 1.00 |  | 1.00 |  | 1.00 |  | 1.00 |  |
|  |  |  |  |  |  |  |  |  |
| Education |  |  |  |  |  |  |  |  |
| High school education or less | 1.71* | [1.10, 2.66] | 1.23 | [0.72, 2.11] | 1.24 | [0.72, 2.13] | 1.64 | [0.91, 2.97] |
| Some college or technical school | 1.80** | [1.19, 2.71] | 1.37 | [0.88, 2.14] | 1.38 | [0.88, 2.16] | 1.42 | [0.90, 2.23] |
| College graduate | 1.00 |  | 1.00 |  | 1.00 |  | 1.00 |  |
|  |  |  |  |  |  |  |  |  |
| Marital Status |  |  |  |  |  |  |  |  |
| Widowed, divorced, separated, or never married | 1.52* | [1.06, 2.18] | 1.11 | [0.71, 1.73] | 1.09 | [0.70, 1.71] | 1.06 | [0.67, 1.68] |
| Married or living with a partner in a marriage-like relationship | 1.00 |  | 1.00 |  | 1.00 |  | 1.00 |  |
|  |  |  |  |  |  |  |  |  |
| Living Arrangement |  |  |  |  |  |  |  |  |
| Rent | 1.54* | [1.01, 2.35] | 1.43 | [0.86, 2.38] | 1.37 | [0.82, 2.30] | 1.34 | [0.78, 2.30] |
| Own | 1.00 |  | 1.00 |  | 1.00 |  | 1.00 |  |
| Other arrangement | 1.91* | [1.05, 3.45] | 2.22* | [1.11, 4.42] | 2.05* | [1.01, 4.13] | 2.38* | [1.15, 4.93] |
|  |  |  |  |  |  |  |  |  |
| Comorbidity Index |  |  |  |  |  |  |  |  |
| 0 Comorbidities | 1.00 |  | 1.00 |  | 1.00 |  | 1.00 |  |
| 1 Comorbidity | 1.50 | [0.92, 2.45] | 1.25 | [0.73, 2.13] | 1.28 | [0.75, 2.18] | 1.18 | [0.68, 2.05] |
| 2 Comorbidities | 2.17** | [1.31, 3.61] | 1.50 | [0.84, 2.67] | 1.49 | [0.83, 2.68] | 1.33 | [0.72, 2.48] |
| ≥3 Comorbidities | 2.34*** | [1.44, 3.81] | 1.23 | [0.70, 2.16] | 1.18 | [0.66, 2.08] | 1.04 | [0.56, 1.92] |
|  |  |  |  |  |  |  |  |  |
| Health Insurance Type |  |  |  |  |  |  |  |  |
| Uninsured | 1.37 | [0.71, 2.63] |  |  | 1.55 | [0.77, 3.13] | 1.51 | [0.74, 3.06] |
| Private | 1.00 |  |  |  | 1.00 |  | 1.00 |  |
| Medicare | 2.94** | [1.54, 5.61] |  |  | 1.88 | [0.86, 4.10] | 1.81 | [0.82, 4.00] |
| Medicaid | 1.26 | [0.66, 2.38] |  |  | 1.12 | [0.51, 2.47] | 1.10 | [0.44, 2.75] |
| Another Type | 1.11 | [0.39, 3.20] |  |  | 1.05 | [0.32, 3.49] | 1.08 | [0.33, 3.54] |
|  |  |  |  |  |  |  |  |  |
| Self-Reported Severity of Symptoms |  |  |  |  |  |  |  |  |
| Mild | 1.00 |  |  |  |  |  | 1.00 |  |
| Moderate | 1.40 | [0.72, 2.70] |  |  |  |  | 2.41* | [1.15, 5.08] |
| Severe | 2.70** | [1.47, 4.98] |  |  |  |  | 3.74*** | [1.89, 7.37] |
| Very severe | 5.75*** | [3.07,10.77] |  |  |  |  | 8.26*** | [3.97,17.23] |
| * p<0.05, ** p<0.01, *** p<0.001 Model 1 is unadjusted. Model 2 is adjusted for sociodemographic variables (sex, age group, race/ethnicity, education, marital status, and living arrangement). Model 3 is adjusted for sociodemographic variables and health insurance type. Model 4 is adjusted for sociodemographic variables, health insurance type, and self-reported severity of symptoms. Models 2-4 are additionally controlled for survey type (online versus phone) and sample. | | | | | | | | |
